# Treatment of Erythrocytes with the 2-Cys Peroxiredoxin Inhibitor, Conoidin A, Prevents the Growth of *Plasmodium falciparum* and Enhances Parasite Sensitivity to Chloroquine

**DOI:** 10.1371/journal.pone.0092411

**Published:** 2014-04-03

**Authors:** Mariana Brizuela, Hong Ming Huang, Clare Smith, Gaetan Burgio, Simon J. Foote, Brendan J. McMorran

**Affiliations:** 1 The Menzies Research Institute Tasmania, University of Tasmania, Hobart, Tasmania, Australia; 2 The Australian School of Advanced Medicine, Macquarie University, Sydney, New South Wales, Australia; University of Melbourne, Australia

## Abstract

The human erythrocyte contains an abundance of the thiol-dependant peroxidase Peroxiredoxin-2 (Prx2), which protects the cell from the pro-oxidant environment it encounters during its 120 days of life in the blood stream. In malarial infections, the *Plasmodium* parasite invades red cells and imports Prx2 during intraerythrocytic development, presumably to supplement in its own degradation of peroxides generated during cell metabolism, especially hemoglobin (Hb) digestion. Here we demonstrate that an irreversible Prx2 inhibitor, Conoidin A (2,3-bis(bromomethyl)-1,4-dioxide-quinoxaline; BBMQ), has potent cytocidal activity against cultured *P. falciparum*. Parasite growth was also inhibited in red cells that were treated with BBMQ and then washed prior to parasite infection. These cells remained susceptible to merozoite invasion, but failed to support normal intraerythrocytic development. In addition the potency of chloroquine (CQ), an antimalarial drug that prevents the detoxification of Hb-derived heme, was significantly enhanced in the presence of BBMQ. CQ IC_50_ values decreased an order of magnitude when parasites were either co-incubated with BBMQ, or introduced into BBMQ-pretreated cells; these effects were equivalent for both drug-resistant and drug-sensitive parasite lines. Together these results indicate that treatment of red cells with BBMQ renders them incapable of supporting parasite growth and increases parasite sensitivity to CQ. We also propose that molecules such as BBMQ that target host cell proteins may constitute a novel host-directed therapeutic approach for treating malaria.

## Introduction

Infections by the malaria parasite, *Plasmodium falciparum*, account for almost one million deaths each year. Efforts to reduce the burden of disease are hampered by the development of drug-resistant strains [1]. Chloroquine (CQ) in particular was once a highly effective and mainstay treatment, but is now virtually useless in most parts of the world. The drug is understood to kill the parasite growing inside the red blood cell by targeting the hemoglobin digestion pathway in the parasite food vacuole. Specifically, the drug inhibits the conversion of heme to hemozoin. The build-up of heme generates free radicals and oxidative molecules, including peroxides, which are toxic to the cell [2]. Many parasite strains throughout the world are now insensitive to CQ treatment due to acquisition of variant drug transporter proteins that expel the drug from the parasite and provide a selective advantage under CQ exposure [3], [4].

Erythrocytes are exposed to high levels of oxidative stress from the continual autoxidation of hemoglobin, which produces O_2_^−^ and H_2_O_2_, and free-radical generation from the lipid-rich membrane and heme. Protection from these damaging molecules is afforded by several enzymatic and non-enzymatic-based systems [5]. Among these are the peroxiredoxins (Prx), ubiquitous thiol-containing proteins that catalyze the decomposition of H_2_O_2_ to water. Three Prx proteins are found in erythrocytes, Prx 1, 2 and 6; Prx2 is by far the most important and is the third most abundant protein in the cell [6]. Prx2 exists as a homodimer. It decomposes H_2_O_2_ via the oxidation peroxidatic cysteine residues (one per monomer), resulting in the formation of cysteine sulfenic acids and subsequent formation of an intermolecular disulfide bond. Reactivation of the enzyme occurs through the action of thioredoxin reductase-coupled thioredoxin, which reduces the intermolecular disulfide bond [7].

The *Plasmodium* parasite also possesses a series of endogenous enzymatic systems to counter the high oxidant environment of the erythrocyte in which it resides, as well as the products of its own metabolism. They comprise of glutathione, thioredoxin and superoxide dismutase; the parasite however lacks a catalase-based pathway (reviewed in [8], [9]). As well as studies demonstrating essentiality of at least thioredoxin-dependent protection [10], [11], these systems may also protect the parasite from the actions of antimalarials that induce oxidative stress. For example, early transcriptional responses to CQ exposure include up-regulation of these systems [12]. Inhibition of glutathione synthesis in particular has also been shown to enhance the effect of chloroquine against *P. berghei* infections in mice [13], [14]. Indeed much work has focused on these oxidant protective systems as potential antimalarial targets (reviewed by [15]).

Recently it was suggested that thioredoxin-dependent consumption of peroxides in the parasite may also be mediated by the host red cell Prx2. Koncarevic and colleagues [16] identified Prx2 inside erythrocytic stage *P. falciparum*, and this has since been confirmed in proteomic-based surveys [17]. The protein is imported from the host cell into the parasite cytosol, where it remains biologically active and enzymatically coupled with *P. falciparum*-expressed thioredoxin. Biochemical assays indicate that approximately half the peroxide detoxification activity in the parasite is derived from Prx2 [16]. Interestingly, under CQ exposure the parasite also up-regulates the import of Prx2 [16], suggesting that not only is the host enzyme important for normal parasite growth, but that it also functions to counteract the toxic effects of CQ.

Here we tested if an available chemical inhibitor of 2-Cys-containing peroxiredoxins, conoidin A (2,3-bis(bromomethyl)-1,4-dioxide-quinoxaline; BBMQ), affects the growth of *P. falciparum* parasites. BBMQ chemically reacts with the peroxidatic Cys residues of peroxiredoxins, forming irreversibly inactive homodimers. BBMQ was previously shown to inhibit human Prx2 in epithelial cells, and the Prx2 homolog of *Toxoplasma gondii*. [18].

## Methods

### *P. falciparum* culture

*P. falciparum* strains 3D7 (chloroquine and mefloquine sensitive), K1 (chloroquine resistant) and W2mef (mefloquine resistant), all gifts from R. Anders and L. Tilley, La Trobe University, Melbourne, Australia, were maintained at between 1 and 10% parasitemia in purified AB+ human erythrocytes in a 1% O_2_/5% CO_2_ atmosphere according to the method of Trager and Jensen [19]. The cell culture medium (CCM) comprised of RPMI 1640 (HEPES, glucose and glutamine-free) supplemented with 1X glutamax, 0.2% Albumax (all from Life Technologies, Australia), 4% pooled AB+ human serum (Invitrogen), 10 mM D-glucose, 25 µg/ml gentamycin, 6 mM HEPES and 0.2 mM hypoxanthine (all from Sigma). “CCM-wash” lacked serum and Albumax. Human red cells were supplied as expired red cell packs by the Australian Red Cross Blood Service (Bloodbank). Ethical approvals to work with the cells were obtained from the University of Tasmania and Macquarie University Human Research Ethics Committees (project numbers H09004 and 5201200714, respectively).

### Drug treatments and parasite growth assays

Parasite cultures were synchronized prior to treatment at either the mature pigmented trophozoite stage (using Percoll gradient sedimentation) or immature ring stage (using two cycles of 5% sorbitol separated by 6 hours). Parasites were seeded in CCM with uninfected red cells at a final parasitemia of approximately 1% and a hematocrit of 2%. CQ and BBMQ (both from Sigma Aldrich, Castle Hill, Australia) were diluted to 5× final concentration in CCM, and added to the prepared parasites at a volumetric ratio of 1∶4. Parasite growth was determined by counting proportions of infected cells in Giemsa-stained blood smears. Percentage growth inhibition was calculated as the proportional difference in growth between treated and untreated cultures. All growth assays were performed at least twice, with duplicate or triplicate culture wells; at least 400 cells were counted for each well.

### BBMQ pretreated red cells (washout)

Uninfected red cells were treated with BBMQ for 24 hours in CCM-wash at 4°C with constant mixing. The cells were then centrifuged, supernatant removed and washed three times in 100× cell pellet volume of CCM-wash. For untreated controls, cells were incubated and washed in CCM-wash without BBMQ under identical conditions. To check the effectiveness of the cell washing protocol, cells were also incubated with an IC_100_ concentration of CQ (100 nM) for 24 hours and similarly washed. A CQ sensitive parasite strain (3D7) grew normally in these cells (data not shown). We also performed experiments that showed that BBMQ treatment had no effect on the morphology or osmotic fragility of red cells (tested up to 25 µM; data not shown). In the CQ treatment experiments, synchronized trophozoites were incubated with the washout cells for 12 hours to allow merozoite invasion to occur, after which CQ was added.

### Isobologram analysis

Different fixed ratios of BBMQ and CQ (both at approximately 5× IC_100_ concentrations) were prepared, serially diluted, added to parasite cultures and growth assays conducted over a 48 hour incubation period. Stock solutions of BBMQ (250 µM) and CQ (0.8 and 40 µM for 3D7 and K1, respectively) were mixed together at the indicated proportions, serially diluted (2-fold) with CCM, and then mixed at a 1∶4 volumetric ratio with parasitized cells (∼1% parasitemia, 2% hematocrit in CCM. Samples were analyzed for growth after 48 hours incubation using YOYO-1 dye staining and flow cytometry [20].

### TUNEL labeling

Thin blood smears prepared from parasite cultures were air-dried, fixed for 30 s in methanol, fixed for 20 min in 1% paraformaldehyde in PBS, washed in PBS, blocked and permeabilized with 1% BSA/0.05% Triton X-100 in PBS, and then labeled with the TUNEL assay. TUNEL assay labeling was performed using the Apo BrdU TUNEL Assay Kit (Molecular Probes, Eugene, OR) using methods described in [21].

### Immunobloting

Mature pigmented trophozoite stage parasitized cells were harvested from parasite cultures using Percoll density gradient centrifugation, and washed three times in PBS. To isolate the intracellular parasites from the surrounding red cell, the washed cells were treated with 0.15% saponin (10× cell volume) for 10 min on ice, and then washed 3 times with 100× volume 0.15% saponin. The parasite pellets were suspended in SDS loading buffer containing 100 mM dithiothreitol (DTT), sonicated (3×30 s), and then heated to 95°C for 5 min. Samples of uninfected red cells were also prepared by addition to SDS loading buffer (with or without DTT and N-ethylmaleimide). Following centrifugation, the soluble fractions (20 µg per lane) were separated on 12% SDS-PAGE gels (Biorad, Australia) under reducing conditions. Following transfer to nitrocellulose membrane, they were blotted with antibodies against human Prx2, hyperoxidized Prx2 (Prx-SO_2/3_) and hexokinase (all from Abcam, Cambridge, MA), diluted in PBS/5% skim milk powder/0.05% Tween-20 overnight at 4°C. After washing (PBS/0.05% Tween-20), membranes were incubated with peroxidase-conjugated secondary antibodies for 4 h at 4°C. Chemiluminescent detection was performed using Immobilon Western Chemiluminescent HRP Substrate reagent (Millipore) and detected using a Chemi-Smart 5000 instrument (Vilber Lourmat).

### Statistical Analysis

P values are calculated using two-tailed t-tests assuming equal variance.

## Results

### Red cell Prx2 is imported into *P. falciparum* parasites and inhibited by BBMQ

**Figure 1:**
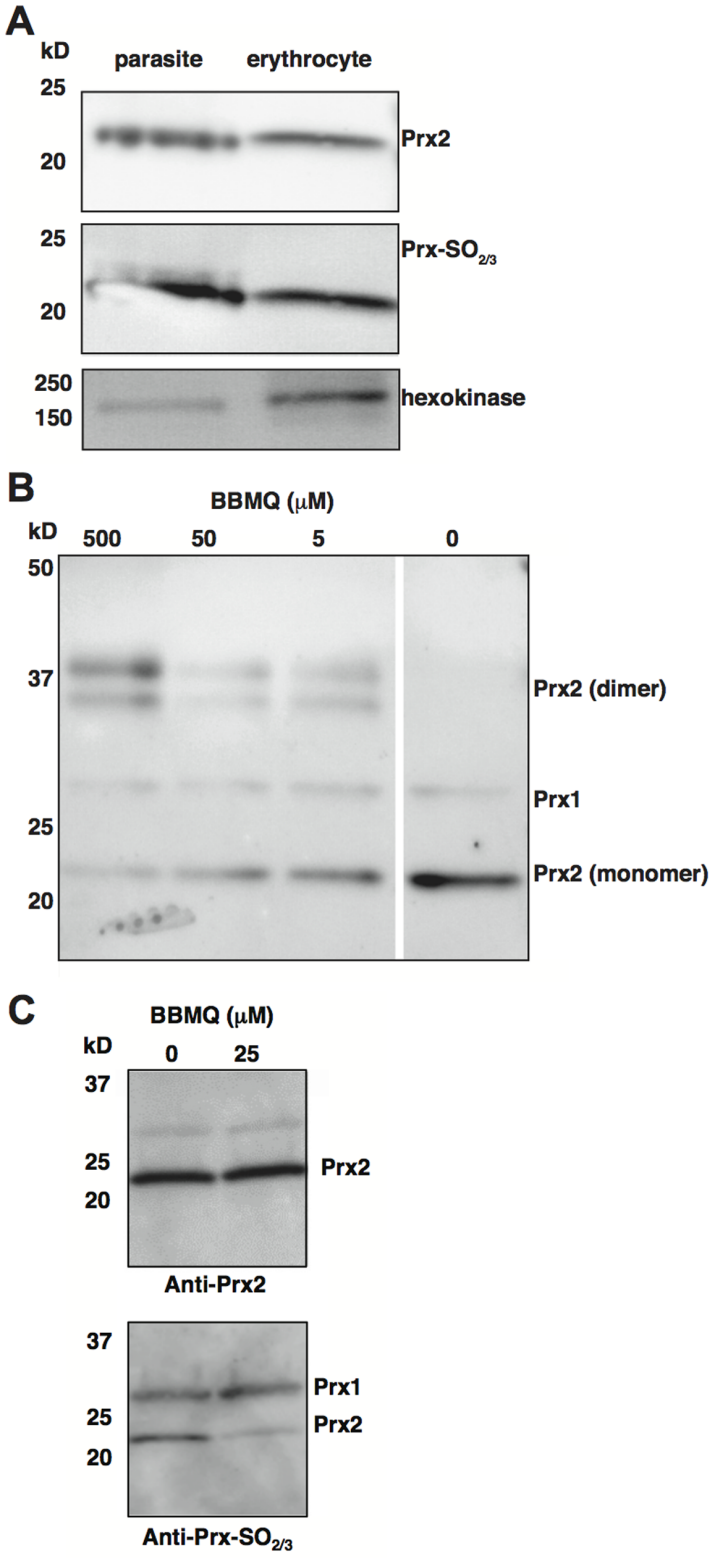
Analysis of erythrocyte peroxiredoxin-2 in *P. falciparum* parasites and the effect of BBMQ treatment. (A) Immunoblot analysis of saponin-purified *P. falciparum* parasites (strain 3D7) and uninfected erythrocytes (separated under reducing conditions) for Prx2, hyperoxidized Prx (Prx-SO2/3) and hexokinase. Equal numbers of cells were loaded in each lane. The presence of human hexokinase indicates the relative level of contaminating red cell proteins in the purified parasite fraction. (B and C) Immunoblot analysis of the effect of BBMQ treatment of human erythrocytes on Prx2 multimer formation using anti-Prx2 antibody (B), and on total and hyperoxidized amounts of Prx2 (C), using the respective antibodies. In (B) proteins were extracted in the presence of N-ethylmaleimide and separated under non-reducing conditions. In (C) proteins were separated under reducing conditions.

We first confirmed the presence of a functionally active Prx2 within intraerythrocytic-stage *P. falciparum* parasites grown in culture. Saponin purified parasites contained enriched levels of the protein, relative to another abundant cytosolic red cell protein, hexokinase (Figure 1A). The protein was also hyperoxidized, indicative of being enzymatically active in the parasite. Next, we showed that BBMQ inhibits Prx2 in human erythrocytes. Similar to the effects of BBMQ on Prx2 previously seen in human epithelial cells [18], exposure of red cells (from expired transfusion packs) to BBMQ induced the formation of high molecular weight forms of Prx2 that were stable under non-reducing conditions in the presence of N-ethylmaleimide. These effects were also concomitant with a reduction of the monomeric protein (Figure 1B). In addition, we analyzed the effect of BBMQ on levels of hyperoxidized Prx2, which is normally detectable in red cells [22]. BBMQ treatment resulted in reduced amounts of hyperoxidized protein, consistent with its ability to covalently bind to the peroxidatic cysteine of Prx2 and block subsequent hyperoxidation (Figure 1C).

### BBMQ is cytocidal against intraerythrocytic *P. falciparum* parasites

**Figure 2:**
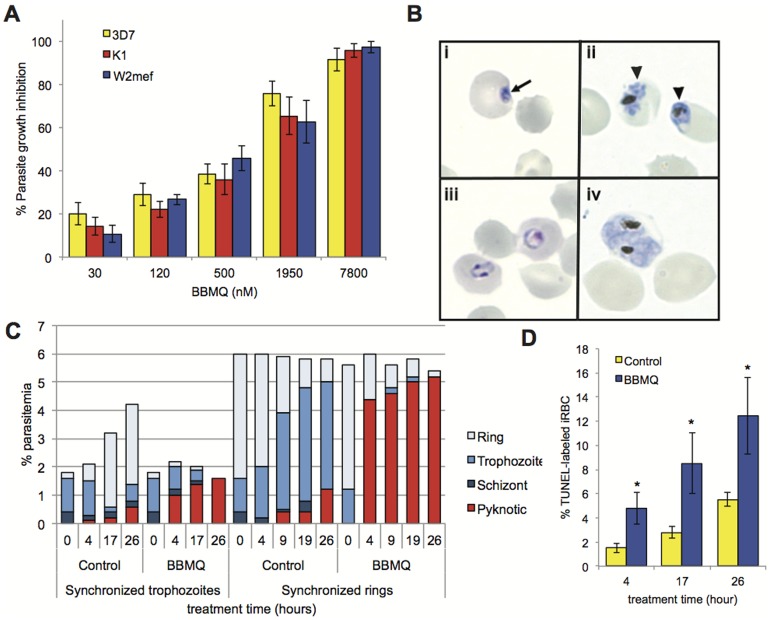
Effect of BBMQ treatment on *P. falciparum* parasite growth. (A) Percentage growth inhibition for three different parasite strains treated with BBMQ. IC_50_ values (± SEM) for the effect of BBMQ were 786±136 nM (3D7), 953±188 nM (K1) and 751±174 nM (W2mef). No significant differences (p>0.5). (B) Photomicrographs of pyknotic or growth-arrested 3D7 parasites following BBMQ treatment. Parasites were synchronized at either the ring stage (panels i and iii) or trophozoite stage (panels ii and iv), and then treated for 12 hours with 25 µM BBMQ (panel i and ii), or left untreated (panel iii and iv). Arrow indicates a parasite of pyknotic appearance; arrowheads indicate growth-arrested trophozoites with condensed and fragmenting nuclei. (C) Percentage of 3D7 infected red cells (% parasitemia) categorized according to parasite developmental stage, following 50 µM BBMQ treatment for indicated times. (D) Percentage of TUNEL-labeled 3D7 parasites following treatment of synchronized ring-stage parasites with 50 µM BBMQ for indicated times. Data shown in (A and D) represent the mean (± SEM) of at least two independent experiments performed in duplicate. * indicates p<0.01.

We tested the ability of BBMQ to inhibit the growth of *P. falciparum* parasites cultured in human red blood cells. The growth of laboratory-adapted strains, either sensitive (3D7) or resistant (K1 and W2mef) to antimalarial drugs, was inhibited by BBMQ treatment in a dose dependent manner (Figure 2A). Similar IC_50_ values were also calculated. BBMQ added at >IC_100_ levels had striking effects on the visual appearance of parasites. There were obvious delays in parasite maturation, as well as parasites with condensed, pyknotic-appearing nuclei, all indicative of stalled growth and/or parasite death (Figure 2B). These observations were quantified in cultures synchronized at either the mature pigmented trophozoite stage or immature ring stage (Figure 2C). The majority of trophozoite-stage parasites treated with BBMQ failed to develop into schizonts and produce new merozoites. BBMQ-treatment of ring-stage cells resulted in a marked reduction in the progression to the more mature forms. In both cases, these effects were obvious after just 4 hours of treatment. Virtually all the cells in the treated cultures were growth-arrested or pyknotic in appearance by the end of the observation period (26 hours). A more objective analysis of this cytocidal effect was conducted using a parasite culture-adapted TUNEL assay [21], which detects fragmented DNA as an indicator of parasite death. BBMQ treatment of immature rings resulted in significantly more TUNEL-labeled parasites compared to untreated cultures after 4, 17 and 26 hours incubation (Figure 2D).

### *P. falciparum* cannot grow in cells pretreated with BBMQ

**Figure 3:**
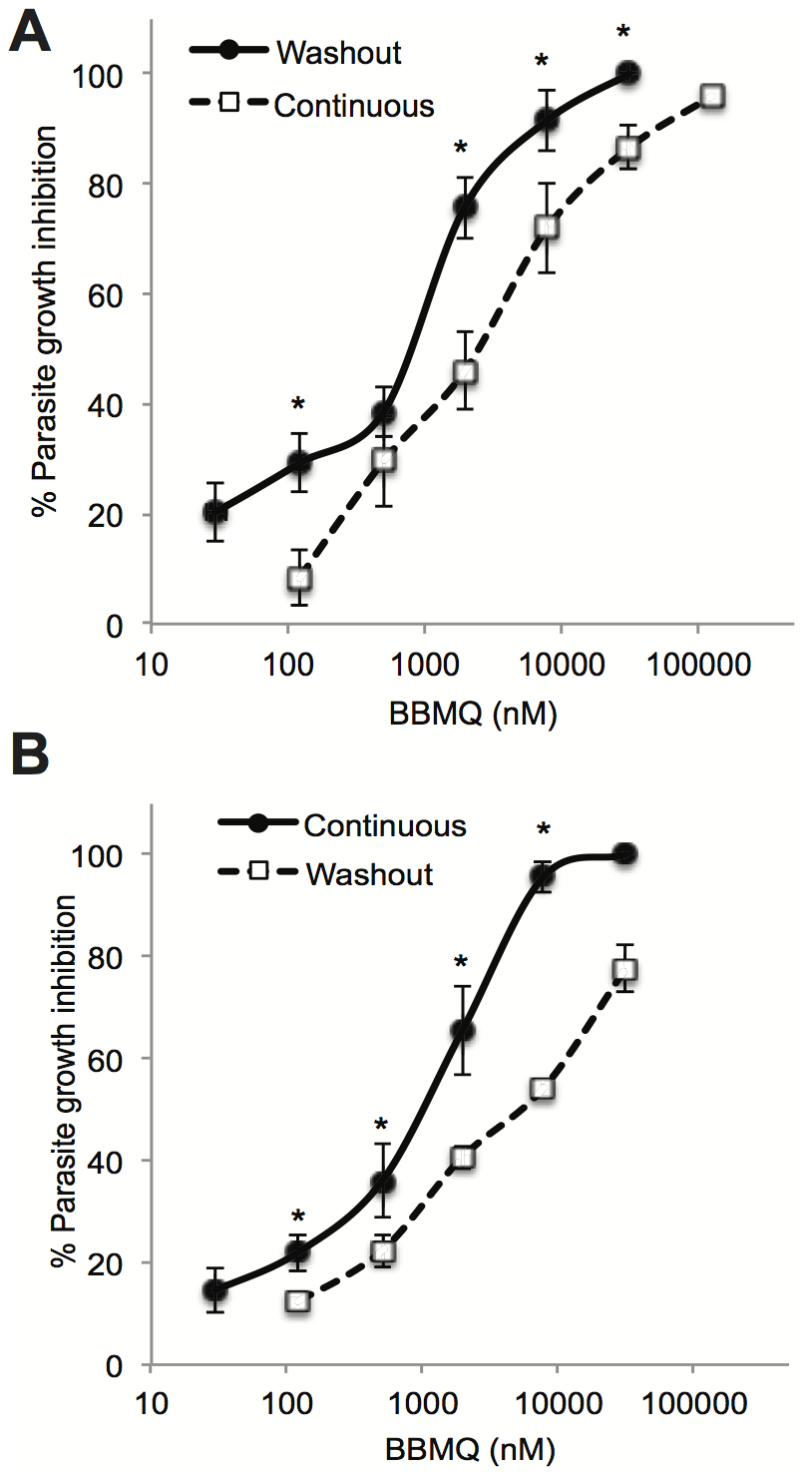
Comparing the effects of continuous and washout BBMQ treatment on *P. falciparum* growth. Percentage growth inhibition of *P. falciparum* 3D7 (A) and K1 (B) using continuous or washout BBMQ treatment. Data represent the mean (± SEM) of at least two independent experiments performed in duplicate. * indicates p<0.01.

Continuously treating the parasite cultures with BBMQ in the above experiments meant that we could not distinguish between effects of BBMQ on host versus parasite proteins. We therefore exploited the irreversible action of BBMQ to determine if specific exposure of the host cell to BBMQ subsequently affected parasite viability. Red cells were pretreated with increasing concentrations of BBMQ and then washed (as described in Methods) prior to parasite infection (called washout). Following addition of purified trophozoites to the washout cells, identical proportions of ring-stage cells were observed after 12 hours incubation, indicating that parasite invasion was unaffected. BBMQ concentrations up to 25 µM were tested without any observable differences on invasion (data not shown). After 48 hours incubation in the washout cells, we noted the appearance of parasites exhibiting signs of growth arrest (at the ring stage), and condensed and pyknotic appearing nuclei similar to observations in the continuously treated cultures. Analysis of cultures after 72 hours also revealed a concentration dependent reduction in the proportion of second-generation rings (and overall parasite growth) (Figures 3A and B). This occurred for both the 3D7 and K1 strains, with similar IC_50_ values (2.2 and 3.9 µM, respectively). Compared to continuous BBMQ treatment, the potency of BBMQ in the washout experiments was reduced three to five fold, suggesting the inhibitory effect may be partly due to BBMQ inhibition of parasite proteins. Taken together, the data indicate that intraerythrocytic development of parasites is prevented by treatment of the red cell with BBMQ. We hypothesize that the compound targets and inactivates host proteins that are normally required by the parasite for its growth.

### *P. falciparum* parasites are more sensitive to CQ in BBMQ-treated cells

**Figure 4:**
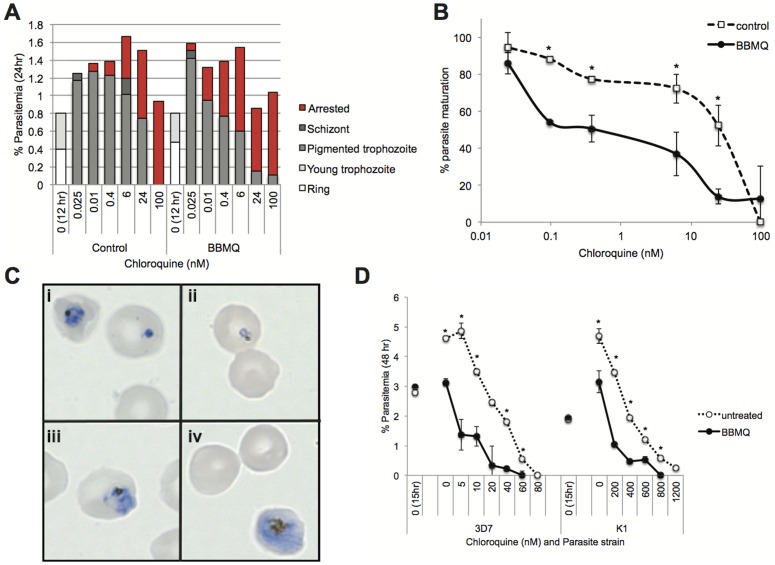
The effects of BBMQ washout treatment on the cytocidal action of CQ. (A) Representative data of the percentage of infected red cells categorized according to parasite stage or growth-arrested appearance after 24 hours incubation with different concentrations of CQ. Red cells were either pretreated with BBMQ, or not treated (control), and then infected with purified trophozoites and allowed to invade and grow for 12 hours. (B) Analysis of the data in (A) with respect to proportions of mature 3D7 parasites (as a function of all parasitized cells) present in cultures after 24 hours incubation. IC_50_ values for the effect of CQ on parasite maturation were 16.3 nM (control) and 1.4 nM (BBMQ). (C) Photomicrographs of growth-arrested 3D7 parasites following CQ treatment for 24 hours (i–iii), and a healthy trophozoite stage parasite observed at the same time point in a control culture (iv). (D) Percentage of red cells containing second-generation ring stage parasites after 48 hours incubation with CQ. IC_50_ values for the effect of CQ on parasite growth with or without BBMQ pretreatment, respectively, were 3D7, 2.3 and 26 nM; K1, 51 and 313 nM. Data in B and D represent the mean (± SEM) of at least two independent experiments performed in duplicate. * indicates p<0.01.

In response to CQ treatment, *P. falciparum* parasites up-regulate the importation of host cell Prx2 [16], presumably to provide the cell with an increased peroxide detoxification capacity and counter the increased oxidative load imparted by the build-up of free heme. We hypothesized that if treatment of the cell with BBMQ inhibits the peroxiredoxin oxidant protection system, we would see an increase in the parasite's sensitivity to CQ. To investigate this, we allowed 3D7 parasites to invade BBMQ washout cells (a sub-inhibitory concentration of BBMQ was used; 250 nM), and then treated with CQ. After 24 hours of CQ treatment, we observed the appearance of malformed and apparently growth-arrested trophozoite-stage parasites, the frequencies of which increased with CQ concentration (Figures 4A and C). Across the CQ titration range, significantly greater proportions of these growth-arrested forms were observed in the BBMQ washout cells compared to control cells (Figure 4B), corresponding to a ten-fold lower IC_50_ for CQ (control, 16 nM; BBMQ washout, 1.4 nM). Similar experiments were conducted using a 48 hour incubation protocol to measure the appearance of second-generation ring-stage parasites. CQ inhibited new ring production in a dose-dependent manner and this effect was significantly enhanced in BBMQ-pretreated cells (Figure 4D); six to ten-fold lower CQ IC_50_ values were calculated (3D7: untreated, 26 nM; BBMQ, 2.3 nM; K1: untreated, 313 nM; BBMQ, 51 nM). Therefore, parasites residing within red cells that have been pretreated with BBMQ are more sensitive to CQ.

**Figure 5:**
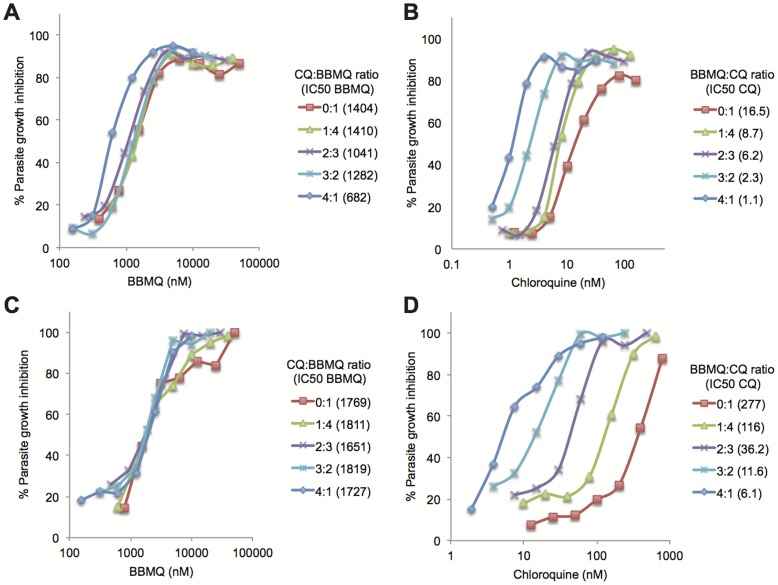
BBMQ-CQ isobologram analysis. Growth inhibition curves for 3D7 (A and B) and K1 parasites (C and D) after 48 hours culture in the indicated concentrations and proportions of CQ and BBMQ. Data represent mean of triplicate wells analyzed in one experiment. The calculated IC_50_ for each compound at each mixture fraction is indicated in the figure keys (nM).

In a second set of experiments we conducted an isobologram analysis of the 3D7 and K1 strains to determine the type of interaction between BBMQ and CQ. Increasing proportions of CQ had little effect on BBMQ IC_50_ values for both strains (Figure 5A and C). However, IC_50_ values for CQ decreased markedly (and in proportion) with increasing ratios of BBMQ (Figure 5B and D). For example, the CQ IC_50_ for 3D7 decreased from 21 nM in the absence of BBMQ to 1.2 nM in the presence of BBMQ added at a 4∶1 ratio. The same 4∶1 BBMQ∶CQ ratio reduced the CQ IC_50_ of the K1 strain more than thirty-fold, and effectively rendered the strain as sensitive to CQ as 3D7. The sum of the fractional IC_50_ values for each strain were approximately one (3D7: 1.01+/−0.18; K1: 1.11+/−0.09), indicating that the growth inhibitory effects of the two compounds were additive. However the additive effect occurred exclusively via the enhancement of BBMQ on CQ activity, and not the reverse. We speculate that this is due to differences in parasite stages most sensitive to each compound. Our observations (Figure 4A) and those made previously [23] show that the cytocidal effects of CQ are not apparent until the later trophozoite stages of development, while BBMQ is cytocidal against early ring-stage parasites (Figure 2C).

## Discussion

In summary, we have shown that treatment of red cells with an irreversible inhibitor of 2-Cys peroxiredoxins, BBMQ, prevents the growth of *P. falciparum* and enhances the sensitivity of the parasite to CQ. BBMQ treatment of red cells resulted in the formation of high molecular weight forms of Prx2 (≈10 µM), which is indicative of enzyme inactivation. This concentration also completely prevented parasite growth; the IC_50_ for the growth inhibition effect was 750–900 nM depending on the parasite strain.

We speculate that the cytocidal action of BBMQ is mediated mainly through the inhibition of red cell Prx2. To exclude the effects of BBMQ on parasite-expressed proteins (*P. falciparum* possesses two 2-Cys peroxiredoxins, TPx1 and TPx2; [9]) red cells were treated with BBMQ and extensively washed prior to parasite infection. These cells supported normal rates of merozoite invasion. However once inside, the parasites failed to grow; DNA synthesis and hemozoin production were impaired, nuclear integrity was lost and viable merozoite production did not occur. The IC_50_ values for BBMQ were lower in the washout experiments compared to continuously treated cultures, indicating that the compound may also affect parasite proteins required for optimal growth.

It is possible that the total redox balance of the erythrocyte is disturbed by BBMQ. However, the morphology and osmotic fragility of cells treated with even high concentrations of the compound (25 µM) was unchanged (data not shown). It is also possible that other erythrocyte 2-Cys containing proteins targeted by BBMQ are also needed for parasite growth. However Prx2 is by far the most abundant enzyme of its class in red cells [6], and no evidence of the parasite importing other red cell 2-Cys proteins has been reported [16], [17]. A direct requirement for red cell Prx2 in parasite growth is also consistent with previous observations that the enzyme is imported by the parasite and remains enzymatically active within the cell [16].

Both early and late-stage developmental forms of the parasite were highly susceptible to exogenous BBMQ treatment, and the effect was rapid. In the case of ring-stage parasites, DNA fragmentation (TUNEL-labeled) and loss of cell integrity were visible within four hours exposure to BBMQ. The cytocidal effect was similarly rapid on trophozoite stage cells. We also note that the pyknotic appearance of the BBMQ-treated parasites is similar to the previously reported effects of the antimalarial drug artesunate. *P. falciparum* parasites treated with artesunate in culture undergo changes resembling an arrest in growth. However upon removal of drug, viable parasites can be recovered, suggesting the drug induces a state of dormancy [24], [25]. We have not tested if the growth of BBMQ-treated cells is similarly recoverable. BBMQ also has a comparably rapid and broad-spectrum activity. Indeed the mechanism of action for artesunate includes the generation of oxidant compounds in the parasite, via an endoperoxide moiety [26]. Molecules with BBMQ-like actions that target oxidant protective mechanisms (in the host cell) may therefore also be highly effective as antimalarials.

Despite the well-reported lack of efficacy and WHO guidelines that discourage its use, CQ continues to be used at high rates in many African countries [27]. This ensures the ongoing existence of CQ resistant strains. Our observations suggest a possible new strategy to treat infections of such strains: target the Prx2 enzyme or other oxidant protective systems in the parasite. Low (sub-inhibitory) concentrations of BBMQ were sufficient to reduce the CQ IC_50_ of normally resistant parasite strains to levels within the normal therapeutic dose window.

We [28] and others [29] have previously proposed a novel host-directed therapy (HDT) strategy for malaria treatment where host proteins, rather than parasite proteins, are targeted. If such host proteins are essential to the parasite, the inhibitors would block parasite growth, *and* avoid potential drug resistance problems. With respect to the possible toxic effects of targeting such proteins with essential functions, it is interesting to note that homozygous null Prx2 mice are relatively healthy and fertile. They do however exhibit a mild anemia and splenomegaly, which is exacerbated if the animals are exposed to oxidant stress-inducing compounds [30], [31]. The action of a compound like BBMQ would have an added advantage in this context by virtue of its irreversible action; short-term treatment would be effective for the life of the red cell (since new protein synthesis does not occur), while synthetically active cells, including erythrocyte progenitors, would restore the pool of functional enzyme. Whether BBMQ or a derivative compound may be safe to administer *in vivo* and produce an antimalarial effect remains to be determined.
